# Tacrolimus Updated Guidelines through popPK Modeling: How to Benefit More from CYP3A Pre-emptive Genotyping Prior to Kidney Transplantation

**DOI:** 10.3389/fphar.2017.00358

**Published:** 2017-06-08

**Authors:** Jean-Baptiste Woillard, Michel Mourad, Michael Neely, Arnaud Capron, Ron H. van Schaik, Teun van Gelder, Nuria Lloberas, Dennis A. Hesselink, Pierre Marquet, Vincent Haufroid, Laure Elens

**Affiliations:** ^1^Department of Pharmacology and Toxicology, Centre Hospitalier Universitaire à LimogesLimoges, France; ^2^Kidney and Pancreas Transplantation Unit, Cliniques Universitaires Saint-Luc, Université catholique de LouvainBrussels, Belgium; ^3^Laboratory of Applied Pharmacokinetics, Children’s Hospital Los Angeles, Los AngelesCA, United States; ^4^Department of Clinical Chemistry, Cliniques Universitaires Saint-Luc, Université catholique de LouvainBrussels, Belgium; ^5^Department of Clinical Chemistry, Erasmus MC-University Medical Centre RotterdamRotterdam, Netherlands; ^6^Department of Hospital Pharmacy, Erasmus MC-University Medical Centre RotterdamRotterdam, Netherlands; ^7^Department of Internal Medicine, Erasmus MC-University Medical Centre RotterdamRotterdam, Netherlands; ^8^Nephrology Service and Laboratory of Experimental Nephrology, University of BarcelonaBarcelona, Spain; ^9^Louvain Centre for Toxicology and Applied Pharmacology, Institut de Recherche Expérimentale et Clinique, Université catholique de LouvainBrussels, Belgium; ^10^Department of Integrated PharmacoMetrics, PharmacoGenomics and PharmacoKinetics, Louvain Drug Research Institute, Université catholique de LouvainBrussels, Belgium

**Keywords:** tacrolimus, kidney transplantation, CYP3A, single nucleotide polymorphisms, population pharmacokinetics, dosage recommendations

## Abstract

Tacrolimus (Tac) is a profoundly effective immunosuppressant that reduces the risk of rejection after solid organ transplantation. However, its use is hampered by its narrow therapeutic window along with its highly variable pharmacological (pharmacokinetic [PK] and pharmacodynamic [PD]) profile. Part of this variability is explained by genetic polymorphisms affecting the metabolic pathway. The integration of *CYP3A4* and *CY3A5* genotype in tacrolimus population-based PK (PopPK) modeling approaches has been proven to accurately predict the dose requirement to reach the therapeutic window. The objective of the present study was to develop an accurate PopPK model in a cohort of 59 kidney transplant patients to deliver this information to clinicians in a clear and actionable manner. We conducted a non-parametric non-linear effects PopPK modeling analysis in Pmetrics^®^. Patients were genotyped for the *CYP3A4^∗^22* and *CYP3A5^∗^3* alleles and were classified into 3 different categories [poor-metabolizers (PM), Intermediate-metabolizers (IM) or extensive-metabolizers (EM)]. A one-compartment model with double gamma absorption route described very accurately the tacrolimus PK. In covariate analysis, only *CYP3A* genotype was retained in the final model (Δ-2LL = -73). Our model estimated that tacrolimus concentrations were 33% IC_95%_[20–26%], 41% IC_95%_[36–45%] lower in *CYP3A* IM and EM when compared to PM, respectively. Virtually, we proved that defining different starting doses for PM, IM and EM would be beneficial by ensuring better probability of target concentrations attainment allowing us to define new dosage recommendations according to patient *CYP3A* genetic profile.

## Introduction

Tacrolimus (Tac) reduces the risk of rejection after solid organ transplantation. However, its toxicities are well known (Kershner and Fitzsimmons, 1996). Consequently, many transplant professionals and pharmacologists have to manage its narrow therapeutic window. Given its highly variable pharmacologic (pharmacokinetic [PK] and pharmacodynamic [PD]) profile (Staatz and Tett, 2004), therapeutic drug monitoring (TDM) is used to individualize Tac dosages and reduce the risks of toxicity and rejection. However, traditional TDM remains a reactive strategy that requires a PK steady state, i.e., approximately 3 days after therapy initiation or dosage change. The delay caused by repetitive dose changes is prohibitive in the early achievement of safe and effective Tac levels (Staatz et al., 2001; Borobia et al., 2009; Richards et al., 2014). Identification of invariable PK biomarkers can help to proactively adjust the dose. However, the identification of useful and relevant biomarkers is only the first step toward therapy individualization. Once the marker is identified, its effect on drug PK variability must be quantified. A population-based PK (popPK) approach can help to model quantitatively the effect of a patient covariate on the drug PK profile in order to simulate the most probable response for a given patient allowing the design of a personalized drug dosage.

Tacrolimus is metabolized in the intestine, in the liver and, to a limited extent, in the kidney by the Cytochromes P450 (CYP) 3A4 and 3A5 enzymes (Staatz and Tett, 2004; Kamdem et al., 2005). It is now universally recognized that a single nucleotide polymorphism (SNP) in the *CYP3A5* gene is associated with approximately a 40 to 50% decrease in Tac clearance (Anglicheau et al., 2003; Hesselink et al., 2003; Haufroid et al., 2004, 2006; Macphee et al., 2005; Elens et al., 2007). Inclusion of *CYP3A5^∗^3/^∗^3* loss-of-function (LOF) allelic status for Tac initial dosage calculation achieves therapeutic levels more quickly (Thervet et al., 2010). However, despite the PK improvement it generates, the clinical benefit in terms of outcome of such a pro-active dosage strategy has not been proven yet but some limitations in study designs have been highlighted (van Gelder and Hesselink, 2010). However, even the PK benefit of a proactive dosage based on *CYP3A5* genotype solely is controversial (Shuker et al., 2016).

Recently, the *CYP3A4^∗^22* decrease-of-function (DOF) allele has been suggested as a good candidate to further refine the Tac starting dose (after adjusting for *CYP3A5* genotype) (Elens et al., 2011, 2013a,b,c,f; Gijsen et al., 2013; Guy-Viterbo et al., 2014; Hesselink et al., 2014; Kuypers et al., 2014; de Jonge et al., 2015a; Tang et al., 2016). However, to our knowledge, only two PopPK studies have examined the combined effects of the *CYP3A4^∗^22* and *CYP3A5^∗^3* SNPs, showing that CYP3A4 DOF can exacerbate the CYP3A5 LOF (Moes et al., 2016; Andreu et al., 2017). The definition of a rationale categorization of the patient into poor (PM), intermediate (IM), and extensive metabolizer (EM) according to these two SNPs has been successfully proposed previously and takes the advantage of being clearly understandable for clinicians and medical staff. The classification is based on the fact that the *CYP3A4^∗^22* allele is associated with a decrease of CYP3A4 function while *CYP3A5^∗^3* is linked to a loss of CYP3A5 expression and that both metabolic defects have synergistic effects. Rationally, the PM cluster contains *CYP3A5^∗^3* homozygotes carrying the *CYP3A4^∗^22* variant; the IM group contains *CYP3A5^∗^3* homozygotes but not carrying the *CYP3A4^∗^22* allele; and EM includes CYP3A5 expressers also not carrying the *CYP3A4^∗^22* allele.

Apart from these functional SNPs, there are a plethora of satellite genes that could affect the function of CYP3A isoenzymes (Werk and Cascorbi, 2014). We describe two of the most promising SNPs among these genes.

Genetic variation in the Peroxisome proliferator-activated receptor a (*PPARA)* gene, a nuclear receptor, was discovered as a novel genetic determinant influencing CYP3A4 activity (Klein et al., 2012). The minor allele of the *PPARA* rs4253728G > A polymorphism has been associated with significantly decreased CYP3A4 expression and activity (Klein et al., 2012; Elens et al., 2013e). This polymorphism might therefore influence the pharmacokinetics of drugs that are primarily metabolized by the CYP3A4 enzyme, such as Tac.

P450 oxidoreductase (POR) is a membrane-bound protein, which is responsible for the transfer of electrons from NADPH to microsomal type II cytochrome P450 enzymes. Liver-specific POR-knockout mice are phenotypically normal but accumulate lipids in the liver and show considerably decreased hepatic drug metabolism (Henderson et al., 2003). Numerous POR missense mutations in humans have been discovered and linked to anarchic steroidogenesis, ambiguous genitalia, and Antley–Bixler syndrome (Huang et al., 2008). In the general population, the 1508C > T SNP (rs1057868; *POR^∗^28*) is the most common variant with a reported minor allelic frequency (MAF) of 30% in the white population. *POR^∗^28* encodes the amino acid variant A503V, which has been associated with differential CYP450 activity (de Keyser et al., 2013; Elens et al., 2013d, 2014). For instance, CYP3A5 expressers carrying one or two *POR^∗^28* alleles have shown a 45 % lower midazolam metabolite conversion and higher Tac dose compared with CYP3A5 expressers without *POR^∗^28* (Elens et al., 2013d).

The first objective of our study is to use a population-based PK approach to simultaneously evaluate the relevance of genotypic and non-genotypic covariates formerly identified as influencing Tac PK. The second objective is to translate our findings into rationale initial dosage recommendations for clinicians that maximize the probability of achieving desired Tac concentrations after the initial dose.

## Materials and Methods

### Patients

The study protocol was approved by the local Ethical Committee (Comité éthique Hospitalo-facultaire of the Saint-Luc Hospital) and all patients provided their written informed consent before taking part in the study.

The previously described study population consisted of 59 cadaveric renal transplant recipients (Elens et al., 2013a). They were prospectively recruited between July 2007 and January 2009 at the Cliniques Universitaires St-Luc (Brussels, Belgium) and followed during their entire hospitalization period as previously described (Elens et al., 2013a).

Briefly, for all patients, immunosuppression consisted of a combination of Tac with mycophenolate mofetil (81%) or mycophenolate sodium (19%) and steroids. A standard steroid tapering schedule was followed (Elens et al., 2013a). The initial Tac dose was calculated according to the bodyweight (bw) of the patient (0.10 mg/kg bw, twice daily) and subsequent doses were adjusted according to Tac concentrations measured just prior to the next dose (C_0_). Tac C_0_ was measured daily during hospitalization. During the 1st week after transplantation, the target Tac C_0_ was 10–20 ng/ml. After this 1st week, this target was reduced to 10–15 ng/ml. For every Tac C_0_ that fell outside the targeted range, the Tac dose was rectified by the clinician. In addition to the daily C_0_ measurement, for all 59 subjects blood samples were collected before and 30 min, 1 h 30 min, 3, 4, 8, and 12 h after administration of the Tac morning dose prior to discharge from the hospital. As previously described, all patients were under concomitant therapies but only 21% of them received a P-glycoprotein inhibitor at a reduced dosage (i.e., atorvastatin and proton pump inhibitors). Furthermore, no CYP3A inducers and/or inhibitors were documented in the medical file, reducing the risk of a clinically significant drug-drug interaction (Capron et al., 2010).

### Blood Sampling and Tacrolimus Quantification

Tacrolimus was measured by a chemiluminescent microparticle immunoassay on the Architect^®^ analyzer from Abbott diagnostics Laboratories (IL, United States). The same assay was used throughout the study. The laboratory participated in the International Proficiency Testing Scheme organized by Dr. Holt in the United Kingdom (Wallemacq et al., 2009).

### Genotyping Analysis

Genomic DNA was extracted from whole blood using the QIAamp DNA Mini Kit (Qiagen, CA, United States). Allelic discrimination analysis was performed for the determination of *CYP3A4^∗^22* (rs35599367C > T, NG_008421.1:g.20493C > T), *CYP3A5^∗^3* (rs776746A > G, NG_007938.1:g.12083G > A), *POR^∗^28* rs1057868C > T (NG_008930.1:g.75587C > T) and *PPARα* rs4253728G > A (NG_012204.1:g.68569G > A) genotype using the TaqMan^®^ (Applied Biosystems, CA, United States) genotyping assays (C__59013445_10, C__26201809_30, C__31052401_10 or C___8890131_30) according to manufacturer instructions.

### Pharmacokinetic Population Modeling

A non-parametric model was developed in Pmetrics^®^. Pmetrics^®^ is a free access Software developed by the Laboratory of Applied PharmacoKinetics and Bioinformatics (LAPK) in Los Angeles CA in the United States. The PK profiles were best described by a one-compartment model with first order elimination. The absorption kinetics was fashioned with 2 distinct but parallel routes of oral absorption, both following a gamma pattern. This model has been previously described and validated in 2 independent cohorts to model immediate and delayed-release form of Tac in lung and renal transplant patients, respectively (Saint-Marcoux et al., 2005, 2010). Details are given in Supplemental Data 1.

For the error model, to weight the concentrations by the reciprocal of their variances in the fitting process, we used a polynomial error of the form SD = 0.0001 + 0.0762 × C(t) - 0.1433 × C(t)^2^ where *SD* is the standard deviation of the measured concentration, and *C(t)* is the measured Tac concentration. The coefficients for the equation were determined by fitting the standard deviations of replicate measured known concentrations to polynomials of 0 to third order, using the study assay. Additionally, a Gamma factor (γ) was used as a multiplier of the assay associated error, so that total noise equaled γ times the SD. We allowed Pmetrics to fit this γ term in the error model with a factor value starting point set at 1.

Model diagnostics included goodness-of-fit of the observed versus predicted plots, minimization of bias and imprecision, satisfactory normalized prediction distribution error (npde) distribution and consideration and the log-likelihood ratio test (-2LL). The log-likelihood ratio test was chosen for the selecting between two hierarchical models. The difference in -2LL of 2 hierarchical models follows approximately a χ^2^ distribution so that a decrease of 3.84 in the -2LL was considered as statistically significant (*p* < 0.05). Briefly, diagnostics of npde distribution is performed by checking whether the shape, location and variance parameters of the distribution correspond to that of theoretical normal distribution. More details about model evaluation through npde can be found in the literature (Comets et al., 2008).

### Covariate Selection

To select potential influencing factors, univariate associations between median Bayesian posterior estimates of PK parameters and the potential covariates were tested. The different covariates tested included the bodyweight, creatinine clearance (Cockroft-Gault formula), the gender, the age, *ABCB1* 3435C > T and 1199G > A SNPs, the *CYP3A4^∗^22* and *CYP3A5^∗^3* alleles solely but also their *CYP3A* combined clusters, the *POR^∗^28* and *PPARa* SNP. When continuous variables were considered, linear regression analyses were performed and scatterplots of median Bayesian posterior estimates versus the covariate tested were drawn. For categorical variables, normalization of the PK parameter distribution was ascertained through logarithmic transformation and ANOVA were performed under the null hypothesis that the means in the tested groups were equal. A *p*-value of less than 0.05 was considered as statistically significant.

After selection of significant covariates in univariate analysis, a covariate model was built using stepwise forward inclusion followed by backward elimination. In the forward inclusion step, all preselected covariate-PK parameter relationships were tested separately. The model with the greatest reduction in -2LL was retained for the next step and all the remaining covariate-PK parameter couples were tested individually in this new model. When no more covariate could be added on the basis of the statistical significance criterion (i.e., Δ2LL > -3.84), the model obtained was regarded as final.

To test the influence of covariates, categorical factors were introduced as follows:

$$\theta_{\text{j}}\text{=}\theta_{\text{jTPV}}\times {\left(\theta_{\text{COVi}}\right)}^{\text{COVi}}$$

whereas continuous variable were allometrically scaled and tested as follows:

$$\theta_{\text{j}}\text{=}\theta_{\text{jTPV}}{\left(\frac{\text{COVi}}{{\text{COVi}}_{\text{mediam}}}\right)}^{\;\theta_{\text{COVi}}}$$

For both equations, 𝜃_jTPV_ is the typical (mean) value of the j^th^PK parameter(𝜃_j_), 𝜃_COV_ a parameter estimated by the model representing the effect of the i^th^covariate (COV_i_) on 𝜃_jTPV_. The categorical covariates were coded as dummy variables.

### Internal Validation

The stability and performance of the model were assessed though Monte-Carlo simulations. A thousand simulated profiles for each subject were created from the final population model parameters using their own set of covariates, dose and sampling schedule. Visual predictive check (VPC), consisting of graphical assessment of simulation results and comparison the data observed, was performed and npde distributions were checked in order to evaluate the quality of the final model.

### Simulation of Dose Regimens

In order to evaluate the suitability of different dosage regimens as a function of the patient’s genetic profile, Monte-Carlo simulations were performed with each tested profile (genotype clusters with different doses) to generate 1000 time-concentration profiles for each dose-genotype combination. The probability of target attainment (PTA) analyses were then performed to evaluate the chance of reaching a defined therapeutic goal for each simulated set of profiles.

### Statistical Analysis

Statistical analyses other than for PK model development were performed using JMP^®^12.2.0 Pro for Windows (SAS Institute Inc., Cary, NC, United States). Baseline characteristics were summarized as mean and the corresponding standard deviation (SD). *CYP3A* genotype clustering was executed as previously established (Elens et al., 2011). Groups were compared using non-parametric tests. To compare two groups, we used the Mann–Whitney *U*-test, and to compare several groups, the Kruskal–Wallis test was applied. For association between categorical data, we used Pearson’s Chi Square test or Fisher’s exact test, as appropriate. In all cases, *p*-values of less than 0.05 were considered statistically significant.

## Results

Baseline characteristics of the patients and genotype frequencies are reported in **Table 1**. The genotype distributions were in accordance with the Hardy-Weinberg principle and with the frequencies reported^1^. In total, considering the *CYP3A* clustering strategy described earlier (Elens et al., 2011), there were 5 patients classified as poor metabolizers (PM = *CYP3A5^∗^3* homozygotes carrying the *CYP3A4^∗^22* variant), 36 as Intermediate metabolizers (IM = *CYP3A5^∗^3* homozygotes not carrying the *CYP3A4^∗^22*) and 18 as extensive metabolizers (EM = CYP3A5 expressers not carrying the *CYP3A4^∗^22* allele).

**Table 1:** Characteristics of the study population.

Characteristics		
Gender (*n*)	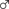	21 (35.6%)
	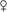	38 (64.4%)
Age (years)		51.9 ± 13.4
Weight (kg)		70.4 ± 13.9
Hematocrit (%)		31.9 ± 5.0
Creatinine clearance at PK course (ml/min)		60.1 ± 20.0
Tac dose before PK course		5.5 ± 2.7
Tac concentrations (ng/ml)	0 min	11.3 ± 4.2
	30 min	19.9 ± 11.7
	1 h30	26.0 ± 11.1
	3 h	22.2 ± 5.5
	4 h	17.4 ± 5.4
	8 h	12.6 ± 4.6
	12 h	10.6 ± 3.8
*CYP3A4^∗^22*	*CYP3A4^∗^1/22*	5 (8.5%)
	*CYP3A4^∗^1/^∗^1*	54 (91.5%)
*CYP3A5^∗^3*	*CYP3A5^∗^3/^∗^3*	41 (69.5%)
	*CYP3A5^∗^1/^∗^3*	14 (23.7%)
	*CYP3A5^∗^1/^∗^1*	4 (6.8%)
*PPARα* rs4253728 G > A	G/G	33 (55.9%)
	G/A	22 (37.3%)
	A/A	4 (6.8%)
*POR^∗^28*	*POR^∗^1/^∗^1*	36 (61.0%)
	*POR^∗^1/^∗^28*	20 (33.9%)
	*POR^∗^28/^∗^28*	3 (5.1%)

*Data are presented either as mean ± standard deviation with Interquartile range in squared brackets for continuous variables or n (% of total) for categorical variables.*

### Development of the Structural Model

A one-compartment model with double gamma absorption route described Tac PK very accurately. Allometric scaling of age and bodyweight did not significantly decrease -2LL. The run converged after 6514 cycles and the final value of the gamma multiplicative factor defining the proportional error model was 0.46. The mean bias between observed and predicted concentrations was not significant and < 1% (-0.11 ± 3.7% and RMSE = 4.5%). The regression analysis of observed versus predicted concentrations yielded a *r*^2^ value of 99.3%. The typical mean PK parameters values (TPV) are reported in **Table 2** (structural model). Inter-patient variability in PK parameters was represented by coefficients of variation ranging from 40 to 80% whereas the correlation between parameters fluctuated from *r* = -0.497 to 0.410.

**Table 2:** Pharmacokinetic parameters of the structural and the final models.

Parameters	TPV Mean	[CI95%]
**Structural Model**
C_0_	2.61	[2.13–3.08]
a_1_	15.70	[8.548–22.91]
b_1_	22.09	[10.30–33.89]
a_2_	16.62	[10.22–23.02]
b_2_	5.40	[0.95–9.85]
r	0.51	[0.46–0.57]
F^∗^A_IV_	21.09	[17.69–24.49]
alpha	1.51	[1.19–1.82]
**Final model**
C_0_	2.94	[2.42–3.47]
a_1_	12.33	[6.25–18.41]
b_1_	20.36	[7.37–33.35]
a_2_	15.19	[9.46–20.91]
b_2_	5.05	[1.02–9.08]
r	0.46	[0.40–0.51]
F^∗^A_IV_	24.52	[20.61–28.43]
alpha	1.52	[1.19–1.85]
𝜃_CY P3A_	0.77	[0.74–0.80]

*C_*0*_ = the model estimated Tac trough level for a theoretical dose of 1000 mg (the real trough level can be calculated by dividing this value by 1000 and multiplying by the patient dose) (*a*_*i*_,*b*_*i*_) = parameters of the gamma distributions, *r* = the fraction of dose absorbed following the first gamma function, F = bioavailability coefficient, A_*IV*_ = initial blood concentration obtained after a bolus IV injection, 𝜃_*CYP3A*_ = parameter representing the effect of the CYP3A covariate on the typical value of the Tac blood concentrations. For more details, see Supplemental Data 1. alpha = elimination parameter.*

### Covariate Analysis

**FIGURE 1:**
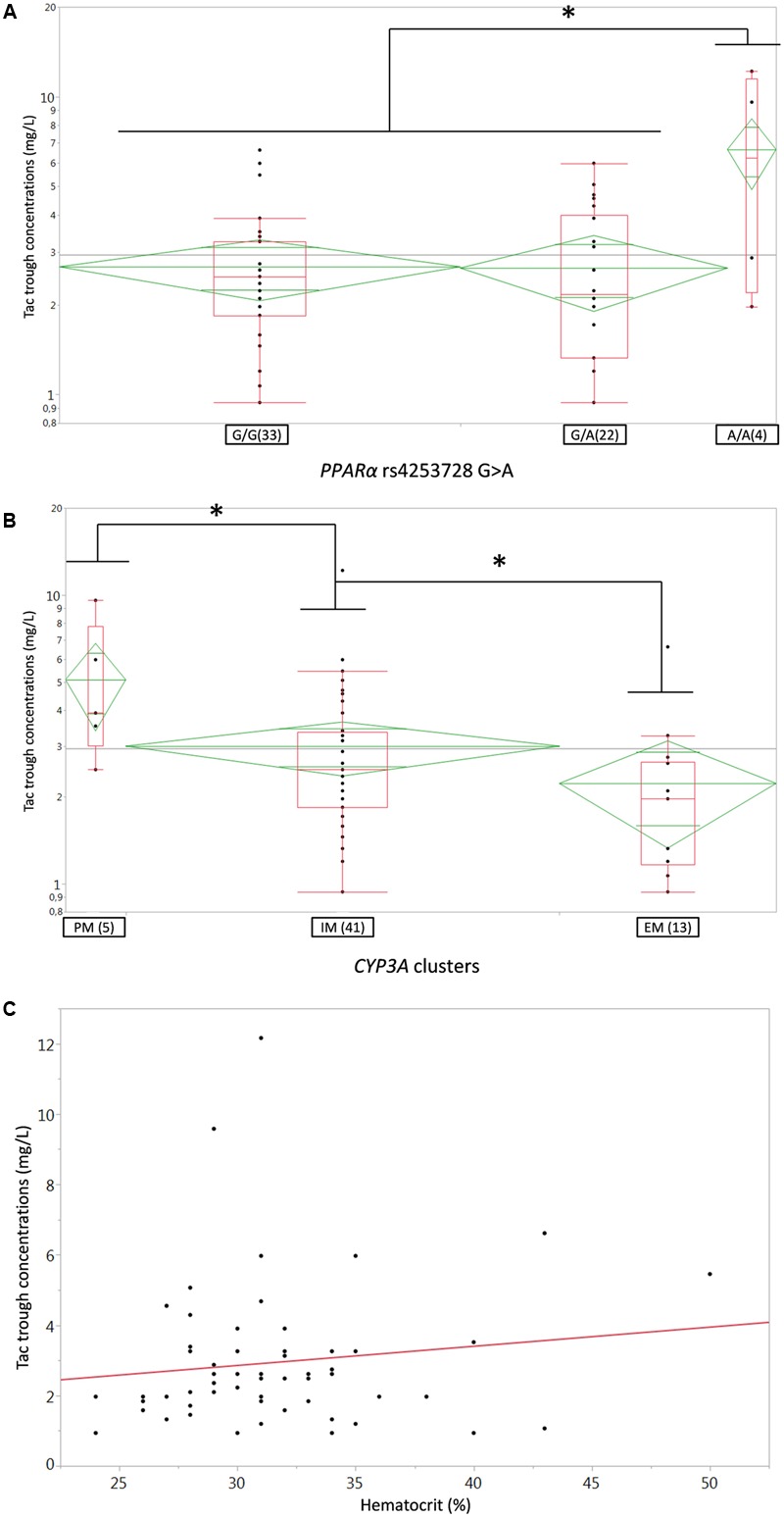
**(A,B)** Box-and-whisker plots of Tac C_0_ (ng/ml) according to **(A)** *PPARa* rs4253728G > A SNP or **(B)** *CYP3A* genotype clusters. The boxes depict the interquartile ranges (IQR) with the bottom and the top of the boxes representing the first (Q1) and third quartiles (Q3), respectively, and the band inside the boxes indicating the medians (Q2), the whiskers link the box with Q1+1.5xIQR and Q3+1.5xIQR and the diamonds represents the means (diagonal) with their respectiveIC95%; **(C)** linear regression plot of Tac C_0_ (ng/ml) on the *Y*-axis versus Hematocrit (%) on the *X*-axis; each dot represents a couple of data for one individual patient, the solid red line represents the fitted linear regression line. PM, poor metabolizer, IM, intermediate metabolizers, EM, extensive metabolizers. ^∗^*p* < 0.05.

As specified in the material and method section, we first tested the influence of bodyweight, creatinine clearance (Cockroft-Gault formula), gender, age, *ABCB1* 3435C > T and 1199G > A SNPs, the *CYP3A4^∗^22* and *CYP3A5^∗^3* alleles solely but also their *CYP3A* combined clusters, the *POR^∗^28* and *PPARa* SNP in the univariate analysis. *CYP3A* clusters (**Figure 1A**), *PPARα* (coded as recessive, i.e., A/A versus G/A+G/G) (**Figure 1B**) and hematocrit (**Figure 1C**) were significantly associated with Tac C_0_ (*p* = 0.006, 0.007, and 0.0011, respectively). These covariates were further retained for testing in the structural model. The other tested covariates were not correlated with any of the Bayesian posterior PK parameters. Consequently, they were not considered for further covariate analysis.

**FIGURE 2:**
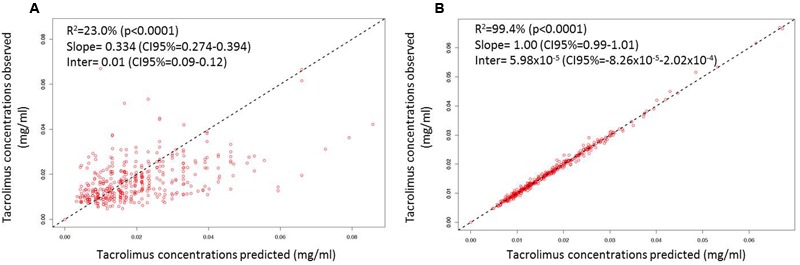
Linear regression of individual observed versus predicted Tac concentrations using **(A)** mean model PK parameter values and **(B)** the means of the individual Bayesian posterior parameter distributions. The dashed lines represent the unity lines.

**FIGURE 3:**
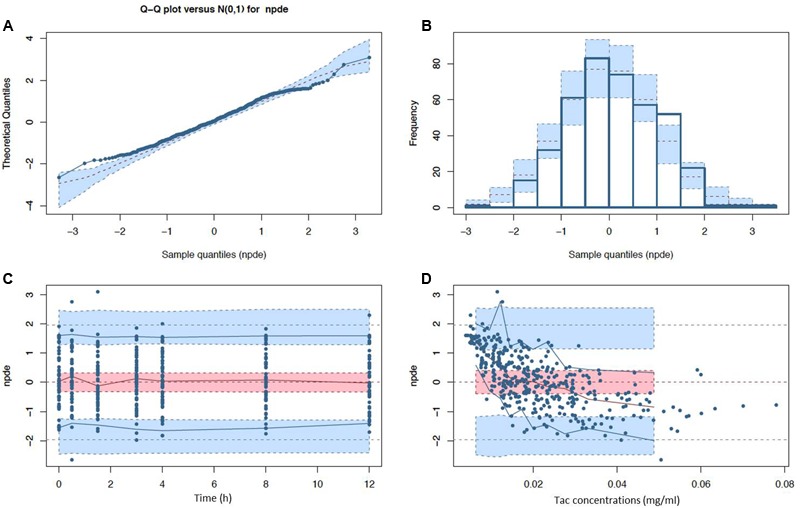
Normalized prediction distribution error (npde) diagnostic plots **(A)** Q-Q plot and **(B)** histogram with expected normal distributions indicated by the dashed lines and light blue boxes (mean and CI_95%_ ranges) and **(C)** npde with respect to post-intake time **(D)** and predicted Tac concentration with observed (solid lines) and expected (dashed lines) npde means (red), 5th and 95th percentiles (blue) with their corresponding CI_95%_ (filled ranges).

**FIGURE 4:**
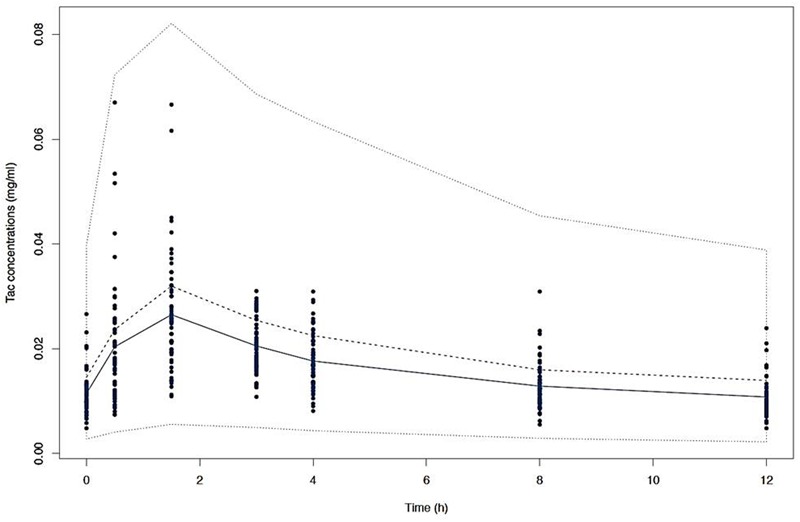
Visual predictive check (VPC) of simulated concentrations (dashed lines) represented by the 10th, 50th, and 90th percentiles versus time with the mean observed Tac concentrations (solid line) and the individual values (dots).

After disjointed forward inclusion, *CYP3A* clusters and *PPARα* improved the model significantly with Δ-2LL of -73 and -4, respectively, whereas hematocrit did not (Δ-2LL = +31). For the next step of forward inclusion with backward elimination, *CYP3A* clustering was chosen as the starting point as it was the covariate with the greatest reduction in -2LL. After inclusion of this covariate, neither *PPARα*, nor hematocrit further improved the fit (Δ-2LL = +184 and +133, respectively). Consequently, only *CYP3A* clustering was retained as a covariate in the final model. The final model converged after 7561 cycles. The 𝜃_CY P3A_ parameter was ascribed to the final output of the model (i.e., the Tac blood concentrations) in the form C(t) = C(t)_TPV_ × (𝜃_CY P3A_)^CY P3A^ where *C(t)* is the Tac blood concentration at time t, *C(t)_TPV_* is the typical value of this PK parameter and 𝜃_CY P3A_ is a parameter estimated by the model representing the effect of the *CYP3A* genotype encoded as a dummy variable. The model-estimated parameters are shown in **Table 2** (final model) and regression plots of observed versus predicted concentrations based on the median population parameters or the median of the individual Bayesian posterior parameter values are represented in **Figures 2A,B**, respectively. Observed concentrations were symmetrically distributed around the predicted values indicating the goodness-of-fit of the model. The gamma error factor for the final model was 0.43. The npde plots resulting from 1,000 simulations for each patient are shown in **Figure 3**. With the exception of a slight negative bias toward negative npde for higher concentrations (**Figure 3D**), our results indicated the absence of any large systematic bias in the model as the prediction errors distribution was centered around 0 with a σ = 1, fitting well with the Normal law (≈[scale=0.5]img001_(μ__=__0,__σ__=__1_), **Figure 3B**). VPC analysis is shown in **Figure 4**. The median of the observed concentrations was close to the median value of the predicted concentrations and all the observations were comprised between the 10th and 90th percentiles of the predicted concentrations.

**FIGURE 5:**
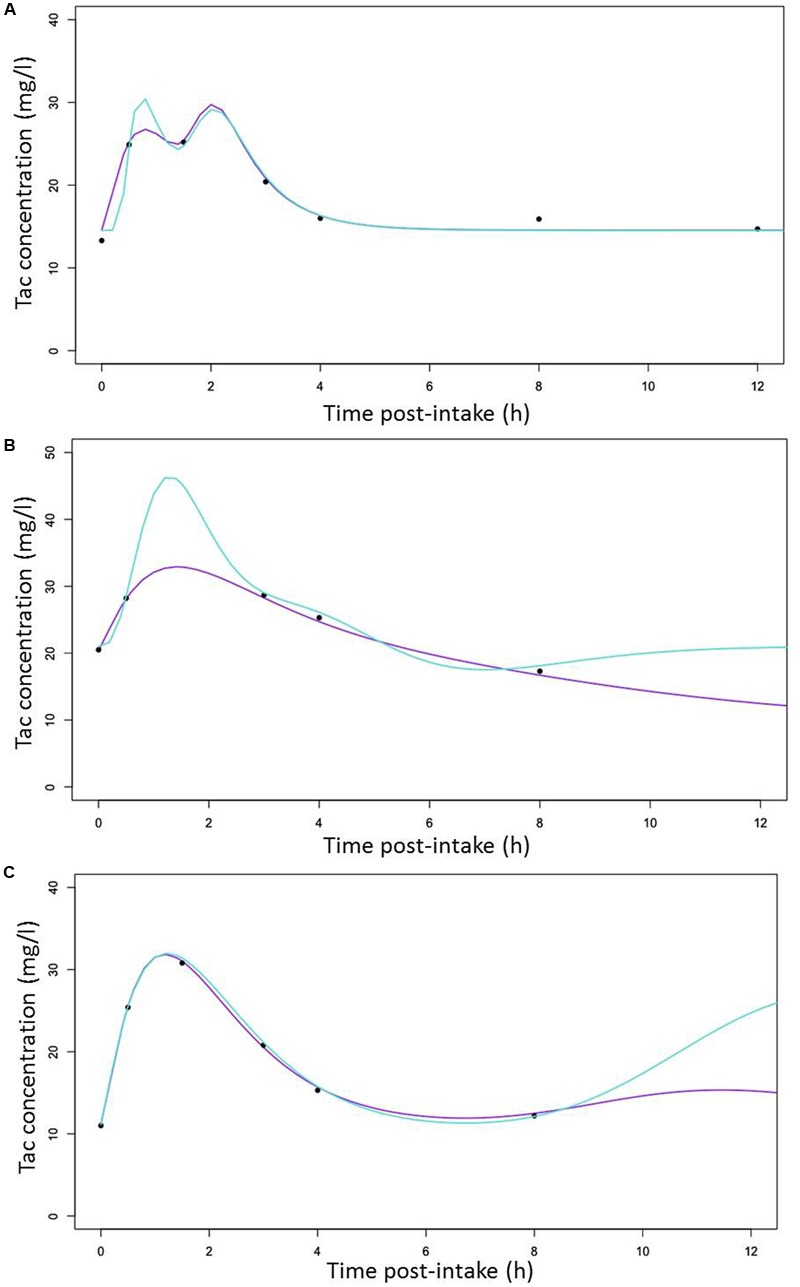
Random selection of Individual predicted Tac concentrations versus time curves (lines) with observed Tac concentrations represented by cross symbols (x) for **(A)** a *CYP3A* poor metabolizers **(B)** a *CYP3A* Intermediate metabolizers **(C)** a *CYP3A* extensive metabolizers with predicted line generated with the structural (turquoise) and the covariate (purple) models, respectively.

The different individual predicted PK profiles were generated for each patient and compared with the observed values. In **Figure 5**, we showed one profile randomly picked in each of the *CYP3A* clusters, generated with the structural (**Figures 5A–C** [turquoise lines]) or the final structural (**Figures 5A–C** [purple lines]) models. Overall, the inclusion of the *CYP3A* clusters as a covariate in the model resulted in improvement of prediction whatever the cluster considered (PM [**Figure 5A**], IM [**Figure 5B**] and EM [**Figure 5C**]).

### Simulations

**FIGURE 6:**
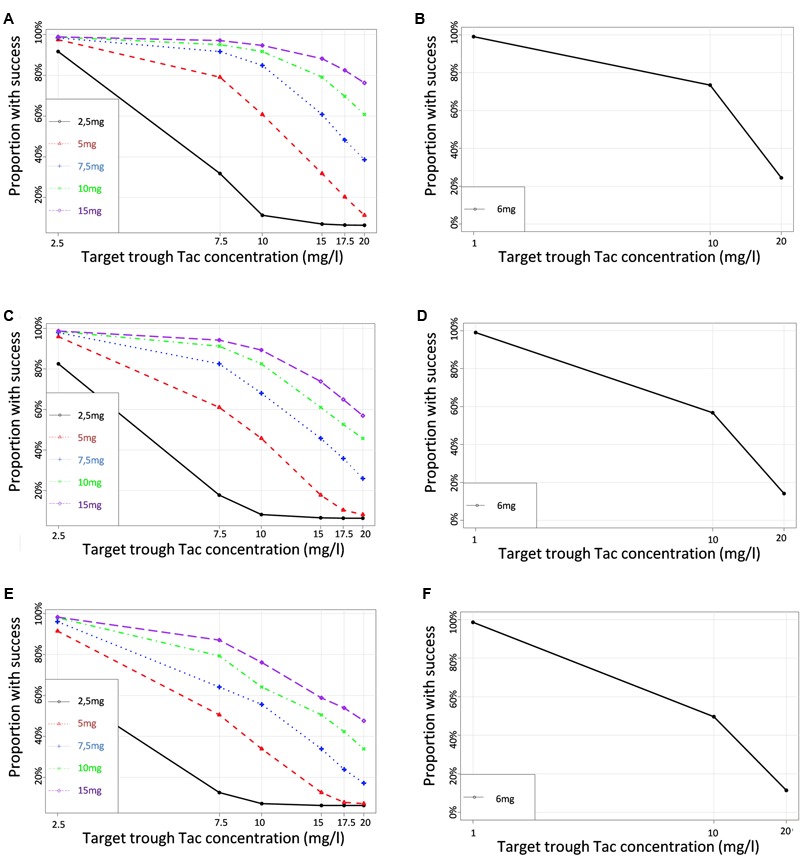
Proportions of simulated patients achieving different Tac C_0_ targets with various dosage regimens in **(A,B)** *CYP3A* poor metabolizers **(C,D)** *CYP3A* Intermediate metabolizers **(E,F)** *CYP3A* extensive metabolizers. Left panels correspond to the simulation performed for a set of virtual dosages, and right panels simulations performed for the actual initial dosage that was really given to the patients.

To define the dose tailored for each genotype group, we performed PTA for each *CYP3A* clusters (PM, IM and EM) with 5 different simulated Tac doses covering the usual doses encountered in clinics (2.5, 5, 7.5, 10, and 15 mg) and 6 different C_0_ targets (2.5, 7.5, 10, 15, 17.5, and 20 ng/ml). Results are presented in **Figures 6A–C** for *CYP3A* PM, IM and EM, respectively, and in **Table 3**. As expected, the PTA increased with higher doses and decreased with higher targets, whatever the *CYP3A* cluster. Considering a ‘therapeutic’ concentration range of 10–20 ng/ml, if we accept a proportion of patients reaching the target of 80% as satisfactory, we can see that simulations predicted adequate doses of 7.5 mg and 10 mg for PM and IM, whereas only 76.1% of EM were expected to reach the threshold of 10 ng/ml with a dose of 15 mg. By contrast, 38.6% of PM treated with a dose of 7.5 mg would reach supra-therapeutic levels of Tac while only 17.2% of EM are expected to attain such high exposure with the same dose. Alternatively, to evaluate the consistency of our PTA predictions with reality, the first administered doses and the corresponding Tac C_0_ were retrieved in the medical records of the 59 patients and used to further simulate the predicted chance to attain a targeted blood level. The overall average initial dose was 6.2 mg and did not differ between the different clusters with 6, 6.2, and 6.5 mg for PM, IM and EM, respectively (*p* > 0.05). With these initial dosages, the actual proportions of patients reaching Tac concentrations above 10 ng/ml on the first measurement (day 1) were 100.0, 52.8, and 25.3% for PM, IM and EM, respectively (*p* = 0.015). The proportions of patients with Tac concentration values > 20 ng/ml on Day 1 were 40.0, 25.0, and 5.9% for PM, IM and EM, respectively (*p* = 0.11). This is approximately comparable to the PTA simulated with our model with respective doses of 6, 6.2, and 6.5 mg for PM, IM and EM (**Figures 6D–F**). Indeed, using these cluster specific doses, Pmetrics predicted that 73.5, 56.7, and 49.6% of PM, IM and EM would have reached 10 ng/ml and 24.5, 14.1, and 11.4% of PM, IM and EM would have at least 20 ng/ml.

**Table 3:** Simulated probability (%) of target attainments (C_0_) according to *CYP3A* genotype and Tac dosage.

C_0_ targets	CYP3A Cluster	Tac simulated doses (mg)				
		2.5	5	7.5	10	15
2.5 ng/ml	PM	91.7%	97.5%	98.4%	98.8%	98.9%
	IM	82.5%	95.9%	97.9%	98.5%	98.7%
	EM	64.1%	91.4%	96.0%	97.9%	98.2%
7.5 ng/ml	PM	31.8%	79.1%	91.7%	95.1%	97.1%
	IM	17.7%	61.0%	82.5%	91.2%	94.2%
	EM	12.6%	50.5%	64.1%	79.3%	87.0%
10 ng/ml	PM	11.3%	60.8%	84.9%	91.7%	94.7%
	IM	8.1%	45.7%	68.0%	82.5%	89.3%
	EM	7.2%	33.9%	55.6%	64.1%	76.1%
15 ng/ml	PM	7.0%	31.8%	60.8%	79.1%	88.2%
	IM	6.5%	17.7%	45.7%	61.0%	73.8%
	EM	6.3%	12.6%	33.9%	50.5%	58.8%
17.5 ng/ml	PM	6.5%	20.3%	48.2%	69.7%	82.5%
	IM	6.3%	10.3%	35.8%	52.6%	64.9%
	EM	6.3%	7.8%	23.9%	42.3%	53.9%
20 ng/ml	PM	6.4%	11.3%	38.6%	60.8%	76.3%
	IM	6.3%	8.1%	25.9%	45.7%	56.9%
	EM	6.3%	7.2%	17.2%	33.9%	47.6%

## Discussion

In classical candidate-gene association studies, the effect of *CYP3A4^∗^22* on Tac PK is well accepted (Elens et al., 2011, 2013a,b,c,f; Gijsen et al., 2013; Guy-Viterbo et al., 2014; Hesselink et al., 2014; de Jonge et al., 2015a; Tang et al., 2016; Andreu et al., 2017). Contrasting with these observations, a number of previous studies have failed to highlight the benefit of introducing *CYP3A4^∗^22* in modeling Tac inter-individual variability through popPK-approaches, with a few exceptions (Shi et al., 2011; Zuo et al., 2013; Zhang et al., 2015; Moes et al., 2016; Andreu et al., 2017). This is not surprising as the majority of studies were performed in Asian populations where *CYP3A4^∗^22* is absent, as it is for individuals of African origins. We clearly showed here that PK prediction can be improved by inclusion of patient *CYP3A4^∗^22* allelic status, particularly via a previously described *CYP3A* cluster classification that takes into account both *CYP3A4^∗^22* and *CYP3A5^∗^3* alleles. Even if it is well accepted that *CYP3A5^∗^3* is the primary factor explaining Tac PK metabolic defect, we showed here that the *CYP3A4^∗^22* PK influence is additive. However, even if the amplitude of the *CYP3A4^∗^22* effect might be comparable to that of *CYP3A5^∗^3*, the *CYP3A4^∗^22* influence is not as statistically significant probably because of the wide PK variability observed among the *CYP3A4^∗^22* carriers. As a consequence, it may also explain why it is not always identified as a significant covariate when considering the few studies reported to date (Shi et al., 2011; Zuo et al., 2013; Zhang et al., 2015; Moes et al., 2016). Another possible explanation is the fact that *CYP3A5^∗^3* completely blunts the CYP3A5 activity whereas for *CYP3A4^∗^22*, some isoenzyme activity remains. An alternative hypothesis is that this lack of statistical reproducibility is due to the lower allelic frequency of *CYP3A4^∗^22* compared to *CYP3A5^∗^3* on the one hand (Bigdeli et al., 2014), and to the fact that CYP3A4 activity is more variable than that of CYP3A5 on the other. Furthermore, the parametric (or semi-parametric) modeling strategy used in previous studies is probably less efficient in detecting inter-individual variability. Indeed, our non-parametric approach benefits from using multiple support points for iterative processing of the data and, as such, each patient is considered as having its own PK parameters distribution and does not rely on the supposition that PK parameters are normally distributed in the general population. This allows better individual prediction and increases the ability to detect differences between individuals. Finally, many confounders can potentially impact on the *CYP3A4^∗^22/CYP3A5^∗^3* effect. In the present study, patients were still hospitalized and environmental influencing factors were potentially better controlled than in ambulatory studies. One can also consider the fact that patients were in the very early period after transplantation where steroids that are known to induce CYP3A activity are still at a quite high dosage. Consequently, steroid induction can have a different impact on CYP3A activity depending on the genetic profile and can boost the difference between the different *CYP3A* clusters making the effect of *CYP3A4^∗^22* even more significant (de Jonge et al., 2015b). Consequently, in previous studies, the effect of *CYP3A4^∗^22* might still be clinically true but just hidden because of the study design and/or modeling method.

Importantly, our study is in total agreement with the conclusions of the study of Andreu et al. (2017) even if the research strategies were dissimilar. Indeed, some important differences in both the design, as well as in the population modeling method render our study different and more robust than the Spanish study. The first difference resides in the study design. In the discovery cohort used to build their model, Andreu et al. (2017), included only 7 patients that were intensively sampled and, as such, providing a complete PK course early after transplantation (day 7). The rest of the samples (98% of the patients) were through levels collected at five different time points with only one collected in the very early post-transplant phase. In our study, a complete PK profile was available for all the patients in the early post-transplant phase. The second main difference resides in the population modeling method. Indeed, in the study of Andreu et al. (2017), Tac concentration-time data were analyzed using a parametric population PK approach with NON-MEM, which assumes that the estimated parameters are normally distributed in the population. However, this assumption might not be true especially if under-represented polymorphic alleles and minority clusters are present in the sample. In non-parametric statistics, no assumptions are made about the underlying distribution of the PK parameters and each patient can serve as a support point for the model-building iterative process and the estimation of PK parameters. As such, instead of obtaining only single-point parameter estimates for the population, one gets multiple estimates, up to one for each subject studied. Consequently, the model comes the closest to the collection of each subject’s exactly known parameter values. Other strengths of the non-parametric approaches include mathematical consistency, good statistical efficiency, and good asymptotic convergence (Jelliffe et al., 2000).

*PPARα* SNPs have been associated with differences in exposure and/or metabolite formation of drugs metabolized through CYP3A. More particularly, *PPARα* rs4253728G > A SNP has been associated with the risk of developing Post-Transplantation Diabetes Mellitus in patients treated with Tac (Elens et al., 2013e). However, our previous investigation failed to explain this increased risk through a PK difference. Here, data suggest that *PPARα* might have an influence on Tac PK, but in a recessive manner. However, in our cohort, only 4 patients were homozygous for the variant allele, among whom 2 were *CYP3A* PM. This obviously renders the statistical power very low and might explain the fact that it was not retained in the final model. Moreover, the effect of *PPARα* is thought to be exerted through an indirect effect on CYP3A4 activity. As a consequence, its effect in *CYP3A4^∗^22* carriers might be lowered and potentially confounded. This information might also partly clarify the fact that *PPARα* SNP was significant only when *CYP3A* cluster was not included in the model.

By simulations of multiple dosing scenarios across the different *CYP3A* clusters, we provide here clear dosage recommendations with well-defined deliverables. With the table presented in this paper, the clinician can use our predictions directly for a given patient. Our model was proved to be efficient to predict the Tac though blood concentration obtained after the first dose administered directly after transplantation. Given our PTA prediction table, the results suggest a starting dose around 0.1 mg/kg bodyweight *b.i.d.* for PM, 0.13 mg/kg bodyweight *b.i.d.* for IM and 0.2 mg/kg bodyweight *b.i.d.* for EM. However, by comparing PTA analysis with observed data, even if we can see that predictions were quite accurate for IM and EM, they were less precise for the PM cluster where our model seems to slightly underestimate the defect caused by *CYP3A4^∗^22*. Consequently, in line with what has been proposed earlier (Haufroid et al., 2006; Thervet et al., 2010; Birdwell et al., 2015; De Meyer et al., 2016) and because of the recent shifts toward lower Tac target ranges (Ekberg et al., 2007), we suggest revising our above advices for PM with a dose of 0.07 mg/kg bodyweight *b.i.d.* These new guidelines are reasonable and in accordance to the original suggestions of Haufroid et al. (2006), whose guidelines have been successfully tested in a randomized clinical trial (Thervet et al., 2010) and further translated in clear recommendations by the CPIC (Birdwell et al., 2015). With the present analysis, we add a slight nuance to their proposal by considering the DOF caused by the *CYP3A4^∗^22* allele. Besides, our innovative classification implies different PM/IM/EM proportions in each group explaining also the subtle modifications we propose here. However, whereas some studies have identified a relationship between Tac exposure and the risk of acute rejection, this has not been a universal finding (Bouamar et al., 2013). This observation clearly questions the clinical relevance of dosage guidelines based on the probability of trough concentration achievement. It has been speculated that currently applied targets saturate the Tac response and that the concentration-effect relationship is reaching its maximum at lower concentrations (Bouamar et al., 2013; Storset et al., 2015). Nonetheless, with the recent trend toward lower Tac target ranges (Ekberg et al., 2007), the need for prediction tools to avoid underexposure will probably increase. Moreover, Tac is known for its exposure-dependent diabetogenic as well as nephrotoxic effects, which reinforces the relevance of such a tool enabling to avoid too high drug exposure.

Our study has, however, some limitations such as the potential confounding effect of co-medications interfering with ABCB1 function. However, as we did not find any influence of *ABCB1* SNPs on the Tacrolimus PK in univariate analysis, it is most likely that these ABCB1 inhibitors will not substantially affect our model, especially given the low dosage of these potentially interacting co-medications. Furthermore, considering these factors would have increased the number of covariates to test and this comes against the general principle of parsimony. Indeed, multiple statistical testing would have amplified the chance of spurious associations leading to over-parametrization of the model. Besides, we did test the potential impact of these co-medications in univariate analysis and no significant associations were found. One second surprising finding is the fact that hematocrit was not retained as a significant covariate in the final model whereas most of previous Tac popPK studies reported a significant effect (Benkali et al., 2009; Woillard et al., 2011; de Jonge et al., 2012; Asberg et al., 2013; Han et al., 2013; Storset et al., 2014, 2015; Andreu et al., 2017). This lack of association can potentially arise from the design of our study. Indeed, given that our patients were still hospitalized and closely monitored, the variability in hematocrit values was not substantial (CV = 15%) and even reduced in PM (CV = 13.7%) and IM (13.6%) after *CYP3A* genotype stratification, providing an explanation on why the effect of hematocrit is no longer observed in a multivariate context when CYP3A genotype is introduced the model. Also, concerning the lack of influence of the age of the patient, as reported in **Table 1**, our population was 51.9 years old on average and it has been described that the age-related PK changes were essentially observed between ages 40 and 50 but that bioavailability was constant at lower and higher relative values in younger and older patients, respectively (Storset et al., 2014). This might explain why we did not find any association between age and Tac PK. Similarly, it has been observed that gender differences in the PK of CYP3A substrates seem to be more pronounced at younger ages compared with in the elderly (Cotreau et al., 2005), providing an explanation why gender was not associated with Tac PK in our cohort.

Finally, it is obvious that our recommendations should be validated through a randomized clinical trial and are open to future amendments with the potential discovery of new biomarkers. For instance, different studies highlighted the importance of P450 oxidoreductase SNPs to explain differences in CYP3A-driven metabolism and in particular the *POR^∗^28* allele. In the present study, we failed to replicate this observation but this could be due to an insufficient statistical power and/or imperfect study design. In particular, the analysis of *POR^∗^28* is complex as it not only depends on the CYP450 activity alteration but also on the inter-protein cooperation which relies on the substrate size and the CYP450 isoform implied.

## Conclusion

We developed here a practical tool to predict Tac exposure after renal transplantation taking into consideration the two patient’s *CYP3A4* and *CYP3A5* genotypes and their linked predicted metabolic phenotypes. We also showed that our model is accurate in predicting the exposure subsequent to the very first Tac dose, indicating that it can be used even when steady state is not yet reached and thus, produces additional information to TDM that can only be initiated profitably when PK steady state is guaranteed, unless a Bayesian approach is used (Asberg et al., 2013). In conclusion, based on our simulations, we predict a different starting dose for each *CYP3A* genotype profile. Therefore, we recommend new starting doses of 0.07 mg/kg bid for PM, 0.13 mg/kg bid for IM and 0.2 mg/kg bid for EM. Subsequently, after therapy initiation, this tool would probably benefit the clinician if used in a Bayesian adaptive control system (Storset et al., 2015).

## Author Contributions

MM, AC, and VH designed the research study; JBW, MN, VH, and LE performed the experiments; JBW, MM, MN, AC, RHvS, TvG, NL, DAH, PM, VH, and LE analyzed the results; JBW, MM, MN, AC, RHvS, TvG, NL, DAH, PM, VH, and LE wrote the manuscript; all authors read and approved the final manuscript.

## Conflict of Interest Statement

The authors declare that the research was conducted in the absence of any commercial or financial relationships that could be construed as a potential conflict of interest.

## Footnotes

1 www.ensembl.org
