# Supplementary material for: Tacrolimus Updated Guidelines through popPK Modeling: How to Benefit More from CYP3A Pre-emptive Genotyping Prior to Kidney Transplantation
Source: Front Pharmacol. 2017 Jun 8;8:358. doi: 10.3389/fphar.2017.00358 (PMC5462973; doi:10.3389/fphar.2017.00358)
Supplement: Supplementary file 1 [file Data_Sheet_1.DOCX]

**Supplemental data 1:** Description of the PoPPK model

In the model, the absorption rate ($v_{abs}\left( t \right)$) was described by the sum of m gamma distributions (here m=2)

$$v_{abs}\left( t \right)=FD\sum_{i=1}^{m=2} r_{i}f_{i}\left( t \right)$$

with

$$f_{i}\left( t \right)=b_{i}^{a_{i}}t^{a_{i}-1}exp\left( -b_{i}t \right)/\Gamma\left( a_{i} \right)$$

where F is the bioavailability coefficient, D the administered dose, Γ the gamma function, (a_i_,b_i_) the parameters of the gamma distributions, and r_i_ the fraction of drug dose absorbed through the i-th route. No independent estimation of the bioavailability was possible because no intravenous data were available for these patients.

The disposition kinetics (*I(t)*), which is defined as the concentration of the drug at time t after a bolus of a unit dose, was best described using a one-compartment model according to the equation

$I\left( t \right)=A_{IV}{exp}^{-kt}$ where A_IV_ is the initial blood concentration obtained after a bolus IV injection.

The convolution product of the absorption rate ($v_{abs}\left( t \right)$) and the disposition kinetics function (*I(t)*) was computed analytically as extensively developed previously and generated the following final expression introduced as the output (*C(t)*) equation in our model file:

$$C\left( t \right)=C_{0}+FDA_{IV}{exp}^{-kt}\sum_{i=1}^{m=2} r_{i}\left[ {b_{i}}/\left( b_{i}-k \right) \right]^{a_{i}}\times P\left[ a_{i},\left( b_{i}-k \right)t \right]$$

where C is the Tac blood concentration at time t and, as the patients where already at pharmacokinetic steady state, C_0_ corresponds to the trough concentration before the input dose. P denotes the incomplete gamma function:

$P\left( n,x \right)=\left[ 1/{\Gamma\left( n \right)} \right]\int_{0}^{x} z^{n-1}{exp}^{-z}dz$

where n is the exponent of the incomplete gamma function, x the independent variable, and z, the integration variable.
